# Swin-HSTPS: Research on Target Detection Algorithms for Multi-Source High-Resolution Remote Sensing Images

**DOI:** 10.3390/s21238113

**Published:** 2021-12-04

**Authors:** Kun Fang, Jianquan Ouyang, Buwei Hu

**Affiliations:** 1 Hunan Meteorological Information Center, Hunan Meteorological Bureau, Changsha 410118, China; k19890823@163.com; 2 School of Computer Science & School of Cyberspace Science, Xiangtan University, Xiangtan 411105, China; hbwslms@163.com

**Keywords:** target detection, Swin Transformer, PReLU, MixUp, remote sensing, multi-source

## Abstract

Traffic port stations are composed of buildings, infrastructure, and transportation vehicles. The target detection of traffic port stations in high-resolution remote sensing images needs to collect feature information of nearby small targets, comprehensively analyze and classify, and finally complete the traffic port station positioning. At present, deep learning methods based on convolutional neural networks have made great progress in single-target detection of high-resolution remote sensing images. How to show good adaptability to the recognition of multi-target complexes of high-resolution remote sensing images is a difficult point in the current remote sensing field. This paper constructs a novel high-resolution remote sensing image traffic port station detection model (Swin-HSTPS) to achieve high-resolution remote sensing image traffic port station detection (such as airports, ports) and improve the multi-target complex in high-resolution remote sensing images The recognition accuracy of high-resolution remote sensing images solves the problem of high-precision positioning by comprehensive analysis of the feature combination information of multiple small targets in high-resolution remote sensing images. The model combines the characteristics of the MixUp hybrid enhancement algorithm, and enhances the image feature information in the preprocessing stage. The PReLU activation function is added to the forward network of the Swin Transformer model network to construct a ResNet-like residual network and perform convolutional feature maps. Non-linear transformation strengthens the information interaction of each pixel block. This experiment evaluates the superiority of the model training by comparing the two indicators of average precision and average recall in the training phase. At the same time, in the prediction stage, the accuracy of the prediction target is measured by confidence. Experimental results show that the optimal average precision of the Swin-HSTPS reaches 85.3%, which is about 8% higher than the average precision of the Swin Transformer detection model. At the same time, the target prediction accuracy is also higher than the Swin Transformer detection model, which can accurately locate traffic port stations such as airports and ports in high-resolution remote sensing images. This model inherits the advantages of the Swin Transformer detection model, and is superior to mainstream models such as R-CNN and YOLOv5 in terms of the target prediction ability of high-resolution remote sensing image traffic port stations.

## 1. Introduction

High-resolution remote sensing images [1] are completely different from ordinary digital images in terms of acquisition methods. Most ordinary digital images come from digital cameras, scanners, and other equipment. The imaging distance is short, the image spatial resolution is high [2], and the imaging parameters are highly arbitrary. Photographic imaging follows the principle of central projection, and the recorded information is mostly the reflection of objects in the visible light band. Remote sensing images are obtained by sensors on aerospace and aviation equipment, and most of the imaging uses the top imaging method, that is, overlooking the ground. This imaging method is far away from the ground, so the spatial resolution is low. In short, high-resolution remote sensing images have the characteristics of small objects of interest, less training data, super-large resolution, diverse target scales, complex image backgrounds, and diverse interference factors. In addition, multi-source high-resolution remote sensing images include satellite images of different series, different orbits, and different inclination angles of meteorological satellites and high-resolution remote sensing satellites. Such images are different in resolution, target angle, target size, and rotation angle [3].

High-scoring remote sensing image transportation port stations (such as airports, ports) are composed of buildings, infrastructure, and transportation vehicles [4]. The detection targets need to gather the characteristic information of nearby small targets, comprehensively analyze, classify, and finally locate. Based on the above problems, this paper summarizes the above problems as the problem of supervised information depth feature aggregation caused by the high-resolution remote sensing image traffic port station target detection representation method. This problem can further affect the model’s ability to locate the traffic port station.

Since AlexNet [5] made a major breakthrough on ImageNet [6], PASCAL VOC [7], and MS COCO [8], CNNs have been leading the research in var IoU visual fields, evolving continuously from the direction of architecture scale, convolutional structure, etc., and have shined in the history of deep learning development. As the basic network, it is of var IoU types, and such visual tasks provide powerful feature extraction and expression, which greatly promotes the prosperity and development of the entire visual field. On the other hand, in the field of natural language processing, a sequence model architecture represented by a transformer has also been born [9]. The use of attention mechanisms to model the long-range dependence of data has greatly improved the performance of the language model. The great success in the field of natural language has allowed scientists to explore the possibility of transformer application in the field of computer vision. Recent studies have shown broad application prospects. The visual transformer architecture of the Swin Transformer constructs the hierarchical feature map of the image on the basis of linear computational complexity. The Swin Transformer constructs hierarchical feature expressions through small image fragments and layer-by-layer neighborhood merging. This architecture allows the model to achieve dense prediction tasks similar to architectures such as U-Net and FPN. The Swin Transformer directly applies a transformer structure to image classification on non-overlapping medium-sized image blocks [10], and combines the mmdet model [11] to perform small target detection. Compared with convolutional networks, it achieves impressive speed accuracy in image classification.

This paper constructs a novel high-resolution remote sensing image traffic port station detection model (Swin-HSTPS). This model uses the Swin Transformer [10] to divide high-resolution pixel images into small pieces of input model training characteristics, incorporate the PReLU [12] activation function and MixUp [13] data enhancement algorithm, enhance image feature information, make up for the lack of training image data set, and strengthen the information interaction of each pixel block. It also avoids the disappearance of the gradient. Compared with mainstream models such as R-CNN [14] and YOLO5 [15], it has natural advantages in processing high-resolution remote sensing images.

## 2. Related Work

The mmdet target detection framework has been integrated into backbone networks such as ResNet [16], R-CNN, and Fast R-CNN to complete classification or target detection [17,18,19]. The target detection model of R-CNN as the backbone network has a good performance in transportation port stations, such as airplanes [20]. This paper is based on the mmdet target model, combined with the detection characteristics of high-resolution remote sensing image traffic port stations, and builds a high-resolution remote sensing image traffic port station detection model with the Swin Transformer [21,22,23] as the backbone network (Swin-HSTPS). Compared with the Swin Transformer detection model, the Swin-HSTPS mainly works in the aspects of the Swin Transformer algorithm improvement, training data enhancement, and overall model parameter adjustment.

The Swin-HSTPS merges the Swin Transformer backbone network algorithm to transfer the high performance of the transformer algorithm to the field of vision [24,25]. This algorithm takes the original image fragment pixels as input, obtains the characteristics of the pixels after encoding, and then transfers the feature expression of the final image. The design lies in the continuous self-attention layer [26,27]. At the same time, the attention module constructed based on the moving window constructs the hierarchical feature map of the image on the basis of linear computational complexity, and then adopts the hierarchical nesting integration to promote the information interaction of the neighboring feature blocks [28].

The Swin-HSTPS combines the “side inhibition” of the PReLU function to activate the neuron characteristics, and performs a nonlinear transformation on the convoluted feature map, improves the information interaction of each pixel block, improves the target detection receptive field, and combines the normalization to cause an “inhibition” effect. From the idea of reducing the average error of the training data, convolution, pooling, and normalization are used to construct a RESnet-like residual network to optimize the forward network [29] of the Swin Transformer algorithm. The Swin-HSTPS integrated into the MixUp hybrid enhancement algorithm enhances the data in the data preprocessing [30] link to make up for the insufficient training image data set to improve the overall network performance, generalization ability, and prediction ability. In addition, the Swin-HSTPS integrated data set is manually labeled to complete automatic format conversion and construct automatic prediction algorithms.

## 3. Target Detection Algorithm Optimization

### 3.1. Model Construction

**Figure 1:**
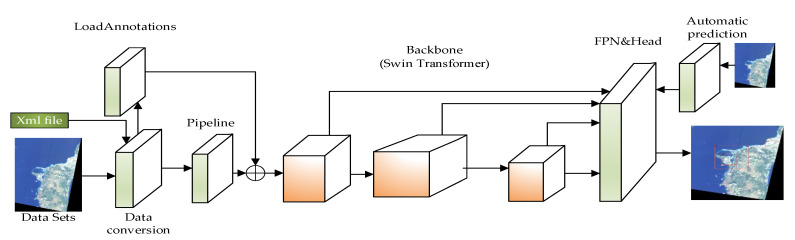
Swin-HSTPS model structure.

This model is based on mmdet, the backbone network is the Swin Transformer, and referring to the Swin-HSTPS parameter settings, a Swin-HSTPS suitable for high-resolution remote sensing image target detection is constructed. The specific Swin-HSTPS structure is shown in Figure 1.

As shown in Figure 1, the overall structure of the Swin-HSTPS model is based on the mmdet model. Compared with the mmdet model, the main improvements of the Swin-HSTPS target detection are as follows:

(1) As shown in the figure above, the pipeline node implements model data preprocessing, data enhancement, and data aggregation for the input of high-resolution remote sensing image training set and test set. In this node, the model is mainly integrated into MixUp (MixUp regularizes the neural network to favor simple linear behavior in between the training example) [13] in terms of data processing. The algorithm is used to enhance the image of mixed types and improve the generalization ability of multi-type target detection data.

(2) In order to adapt to high-resolution remote sensing images and improve the average precision of multi-target detection, this model uses the Swin Transformer algorithm as the backbone network to optimize the forward network of the Swin Transformer algorithm. One structure is similar to the ResNet network to complete the image segmentation process. During the segmentation process, more available features are extracted, and the information interaction of each pixel block is increased, which solves the problem of gradient dispersion when the Sigmoid network is deeper, and slows down the disappearance of the gradient.

(3) The Swin-HSTPS target detection model network model type uses Cascade R-CNN. The number of input 4 stage channels are [128, 256, 512, 1024], respectively; the number of output feature layer channels is 256; and the network dimension is set to 128.

(4) Establish a label data conversion algorithm, convert xml files into json files, and build automated batch prediction algorithms.

(5) According to the generated training model, design an automatic prediction algorithm to predict multi-source remote sensing satellite images in batches, reduce a single budget, and realize the automatic operation of the algorithm from the three stages of testing, training, and prediction.

In short, the Swin-HSTPS model mainly reconstructs the target detection network model based on the Swin Transformer by improving the data preprocessing stage, image segmentation, prediction, and parameter adjustment. At the same time, the label data conversion is constructed to complete the processing of the training set, the prediction set, and the test set.

### 3.2. Incorporate MixUp Hybrid Enhancement Algorithm

Data augmentation is at the core of all successful applications of deep learning, from image classification to speech recognition. In order to improve the generalization ability of multi-source remote sensing image data, this paper will incorporate data enhancement algorithms to process high-resolution satellite remote sensing images. Data enhancement mainly uses operations such as rotation, translation, cropping, resizing, flipping, and random erasing to complete the enhancement operation on the training data set, so as to make up for the lack of training image data set, achieve the purpose of expanding the training data, and improve the generalization ability of the data set.

MixUp is an algorithm used in computer vision to perform mixed enhancement of images. It establishes a linear relationship conversion between data enhancement and supervisory signals to generate a powerful regularizer that can improve generalization capabilities. It can mix images between different classes to expand the training data set. In terms of data enhancement, MixUp has obv IoU advantages over ERM in data sets such as ImageNet-2012 and CIFAR-10. The main theoretical principles of MixUp are as follows:

The Swin-HSTPS inherits the MixUp data expansion method, that is, the linear interpolation method is used to obtain new extended data, and the new training samples and labels are constructed in the linear interpolation method. The principle formula of MixUp is as shown in (1) and (2) [13]:

(1) $$\overset{ˇ}{x}={\mathrm{ax}}_{i}+\left(1-a\right)\;x_{j}$$

(2) $$\overset{ˇ}{y}={\mathrm{ay}}_{i}+\left(1-a\right)\;y_{j}$$

Among them, the two data pairs $\left(x_{i},{\;y}_{i}),(x_{j},{\;y}_{j}\right)$ in the Equations (1) and (2) are the training sample pairs (training samples and their corresponding labels) in the original data set and a~Beta(α,β) is the mixing coefficient calculated from the beta distribution with the parameters α and β.

In order to find the best suitable Swin-HSTPS model for training high-resolution remote sensing images (α, β), select Beta Distribution as (5, 1), (2, 5), (1, 3), (0.5, 0.5) and (2, 2) and other commonly used (α, β) parameters are trained in the Swin-HSTPS model for high-resolution remote sensing images. The specific experimental results are shown in Table 1.

As shown in Table 1, this model has undergone several iterations of training experiments and found that when α = 0.5 and β = 0.5, the average accuracy is 85.3%. Therefore, in the Swin-HSTPS model, when the value of (α, β) is (0.5, 0.5), the training effect is the best.

The target detection model is linear when processing multi-source high-resolution remote sensing image samples and the area between the samples, and the interaction between the samples is weak, and the generalization ability is insufficient in the entire high-resolution remote sensing image. The Swin-HSTPS adds the MixUp mixed enhancement algorithm after data input, and the high-resolution remote sensing image undergoes a series of processes such as rotation, translation, cropping, resizing, flipping, and random erasing, which can express perceptual targets in different forms, such as different transformations of angles, pixels, etc., while linearly transforming the decision boundary from one class to another, which provides a smoother uncertainty estimation. In the original MixUp, the average performance of two neural network models are trained with MixUp and ERM on the CIFAR-10 dataset. The two models have the same structure, use the same training process, and are evaluated on the same sample randomly sampled from the training data. The model trained with MixUp is more stable in predicting the data between the training data.

**Figure 2:**
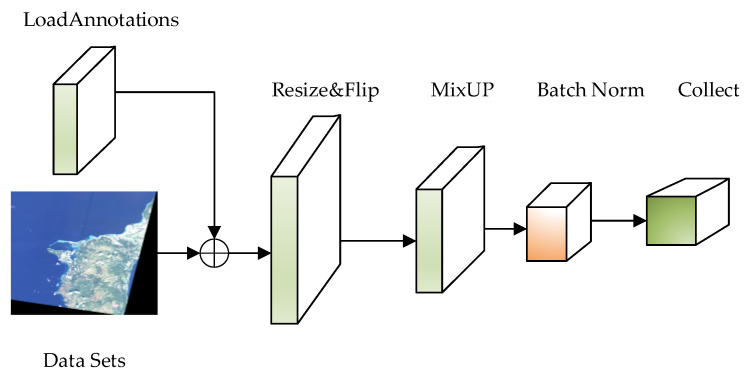
Swin-HSTPS model pipeline process.

After the Swin-HSTPS integrates the MixUp hybrid enhancement algorithm into the pipeline, experiments show that the training model has stronger generalization ability, and the training model has higher average multi-target detection accuracy. At the same time, the discrete sample space is continuous to improve the smoothness in the neighborhood, to make up for the lack of a training image data set. The pipeline flow sequence running process is shown in Figure 2.

As shown in Figure 2, this process loads the data set and label file, randomly flips the image, and incorporates the MixUp algorithm to mix and enhance the data between different classes, thereby expanding the training data set, improving the generalization ability of the data, and enhancing image feature information. Finally, the image size will be readjusted, and the processed data will be input into the backbone network for learning through a series of process operations such as normalization and collection.

### 3.3. Join PReLU Activation Function

The ReLU activation function is a linear correction, and its function is to make it equal to 0 if the calculated value is less than 0, otherwise it keeps the original value unchanged. Using ReLU activation function saves a lot of calculation in the whole process. For the deep network, when the sigmoid activation function is propagated back, the gradient disappears easily, and the training of the deep network cannot be completed. The ReLU activation function will not have a tendency to saturation, and there will be no particularly small gradients. The ReLU activation function will make the output of some neurons be 0, which causes the sparsity of the network and reduces the interdependence of parameters, effectively avoiding the over-fitting problem [31].

In order to improve the generalization ability of the Swin-HSTPS model, it is necessary to construct a residual network similar to ResNet to improve the fit of the Swin-HSTPS model. A very small number of parameters are added to the ReLU activation function, which is compared in the similar ResNet residual network commonly used activation function and parametric rectified linear unit (PReLU) activation function [12] that is widely used in deep learning and machine learning. The PReLU activation function adds a linear function to retain the negative input. The principle of the PReLU activation function is shown in Equation (3).

(3) $$f\left(x_{i}\right)=\{\begin{array}{ll} x_{i}, & x_{i}>0\\ a_{i}x_{i}, & x\leq 0 \end{array}$$

Here $x_{i}$ is the input channel of the ith nonlinear activation function $f\left(x_{i}\right)$, and a_i_ is the slope coefficient of the balance negative part. The subscript i in $a_{i}$ indicates that the non-linear activation is allowed to transform on different channels. When $a_{i}=0$ becomes ReLU, $a_{i}$ is a learnable parameter, such as Equation (3). Equation (3) can be written as Equation (4):

(4) $$f\left(x_{i}\right)=\max\left(0,x_{i}\right)+a_{i}\min\left(0,x_{i}\right)$$

In order to verify that the PReLU activation function has a better effect on high-resolution remote sensing images than the ReLU activation function, the model is iteratively trained at ai = 0, 0.25, 0.4, 0.45, 0.5, and 0.6. The training results are shown in Table 2.

It can be seen from the above table that when a_i_ = 0 and IoU = 0.5, the average accuracy of the model is 66.2%, and the average accuracy is the lowest. When a_i_ = 0.45 and IoU = 0.5, when applying Equations (3) and (4), the average accuracy of the model is 85.3%, so the model has been trained for multiple iterations, and when a_i_ = 0.45, the average accuracy is the highest.

**Figure 3:**
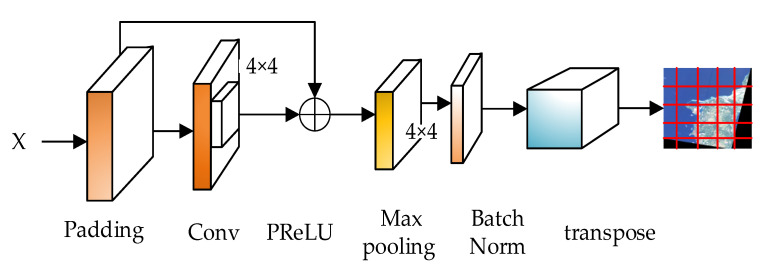
ResNet-like residual network structure.

The ResNet network refers to the VGG19 network, and has been modified on the basis of it, and the residual unit is added through the short-circuit mechanism, as shown in Figure 3. The change is mainly reflected in the direct use of stride = 2 convolution for downsampling in ResNet, and the replacement of the fully connected layer with the global average pool layer. An important design principle of ResNet is: when the feature map size is reduced by half, the number of feature maps doubles, which maintains the complexity of the network layer.

The model backbone network in this paper is improved based on the Swin Transformer model. The original Swin Transformer model algorithm uses the softmax activation function to complete image classification, and the softmax has a gradient disappearance. This model adds the PReLU activation function to the forward network to construct a similar resnet residual network. The nonlinear activation is transformed on different channels, and the information interaction of each pixel block is strengthened, so that the convergence speed of the model is maintained in a stable state, and the gradient is relieved. It disappears quickly to prevent the risk of over-fitting, reduce the depth of the network, improve feature learning, enhance the scalability of its image, and improve the generalization ability. It is similar to the ResNet residual network as shown in Figure 3.

As shown in Figure 3, after the high resolution remote sensing satellite image is input, the large resolution image is segmented, and the convolution kernel and the convolution with a step size of 4 × 4 are performed on the original features, so that there will be no information fusion between different pixel blocks; that is, encode the pixel block, and then use the PReLU function to activate the non-linear transformation of the convolved feature map. After the transformation, the feature information is subjected to a max pooling, and the pooled feature information is normalized, in dim = 2 and dim tile the tensor on the dimension = 3, and then transpose the last two dimensions to form the final segmented feature map output.

### 3.4. Evaluation Index of Detection Algorithm

Target detection of each high-scoring remote sensing image contains many different types of objects. After the target is classified and located through the target detection model, the actual detection performance of the algorithm is evaluated. The Swin-HSTPS follows the mmdet target model evaluation system, which is the Microsoft COCO evaluation system. The average precision and average recall rate in the Microsoft COCO evaluation system reflect the important indicators of the superiority of the model. At present, the Microsoft COCO evaluation system is very popular in the field of computer vision target detection [32]. The main evaluation indicators are as follows:

(1) Cross-pair ratio

Intersection over union (IoU) is commonly used in target detection algorithms to evaluate the degree of overlap between the detection frame and the real frame, that is, the ratio of the intersection of the two rectangular frames to the unity. Generally, a fixed threshold (IoU threshold) is set for the determination of the detection frame. Generally, when the value of IoU is set to be greater than 0.5, it is considered that the target is successfully detected.

(2) AP (average precision)

Precision and recall are the most commonly used evaluation indicators for target detection. The higher the AP, the stronger the predictive ability. The calculation formulas for body detection accuracy and recall rate are shown in (5) and (6), respectively:

(5) $$P=\frac{\mathrm{TP}}{\mathrm{TP}+\mathrm{FP}}$$

(6) $$R=\frac{\mathrm{TP}}{\mathrm{TP}+\mathrm{FN}}$$

TP, TN, and FP are based on the confusion matrix, which counts the number of different detections. TP (true positive) represents the number of targets that were correctly detected, and FP (false positive) represents the number of targets that were not targets but were erroneously detected as targets. FN (false negative) represents the number of targets that are targets but have not been detected [32].

Taking P as the horizontal axis and R as the vertical axis, the PR curve can be obtained. Average precision (AP) averages the precision value on the PR curve [32]. The formula is as shown in (7):

(7) $$\mathrm{AP}=\int_{0}^{1}p\left(r\right)\mathrm{dr}$$

(3) AR (average recall)

AR refers to the maximum recall (maximum recall) of a given fixed number of test results in each picture, averaged over all IoU and all categories.

## 4. Results and Discussion

### 4.1. Data Sets and Processing

High-resolution remote sensing image data are used in the training, testing, and prediction of target detection methods. This article uses AI competition data, GF-2 satellites, GF-5 satellites, FY-4 satellites, and other remote sensing data. Each sheet is marked as an xml file using the labelImg tool [33]. In this experiment, two types of data labeling for airplanes and ports are completed. The xml file is converted into a json file through a conversion algorithm, which conforms to the coco target detection label data format. At the same time, the training remote sensing data are automatically divided into a training set and a test set at a ratio of 9:1, and a training set image set, a test set set, a training set json label, and a test set json label are established.

For this study, the data set is about 80 GB, including about 61 GB of training data and about 19 GB of prediction data. There are a total of 300 selected remote sensing image training data sets; the size of a single image ranges from 20 MB to 1.5 GB and the image resolution ranges from 2500 × 2300 to 19,100 × 32,000 pixel. In addition, there are about 200 selected prediction data sets, with a single sheet ranging from 20 MB to 1.4 GB and the image resolution ranging from 27,100 × 18,000 to 2100 × 2300 pixel.

### 4.2. Model Training Results

This training uses two GeForce RTX 3090 graphics cards, CUDA 11.1, CuDNN 8.0.5, and other basic computing frameworks to complete the experiment. Each experiment was trained in 24 batches. Complete Swin Transformer, Swin-HSTPS, R-CNN target detection model and YOLOv5 target detection model training. The following discussion analyzes the experimental data from different angles.

1. Average precision and performance comparison experiment

After R-CNN, the Swin Transformer, Swin-HSTPS, and YOLOv5 are trained, the parameters, calculation amount, average precision and other indicators are compared. The specific indicators are shown in Table 3.

Table 3 shows the comparison of high-resolution remote sensing images in R-CNN, Swin Transformer, Swin-HSTPS, and YOLOv5 four target detection model-training-related indicators. It can be seen that when the IoU is 0.5, the average precision of the Swin-HSTPS model reaches 85.3%. The average precision of the Swin Transformer model is 77.1%, and the average precision of the Swin-HSTPS is about 8% higher than that of the Swin Transformer model. It is also about 27% higher than the R-CNN model and about 18% higher than that of YOLOv5; when IoU is At 0.75, the average precision of the Swin-HSTPS model reached 49.3%, while the average precision of the Swin Transformer model was 47.5%. The average precision of the Swin-HSTPS model was about 2% higher than the Swin Transformer model and about 9% higher than the R-CNN model. From the above results, it can be seen that the average precision of the Swin-HSTPS is significantly better than the R-CNN, Swin Transformer, and YOLOv5 in high-resolution remote sensing image target detection. In addition, the difference between the Swin-HSTPS and the Swin Transformer in terms of FLOPs computing power is only 0.003 G, and the two models cost the same amount of calculation. From the #param indicator, the Swin-HSTPS and Swin Transformer use similar parameters. From the comparison of indicators in Table 1, it can be seen that the Swin-HSTPS has a better advantage in the average precision of remote sensing images, which is better than the Swin Transformer model. At the same time, the amount of calculation, parameters, and complexity are similar to the Swin Transformer model.

**Figure 4:**
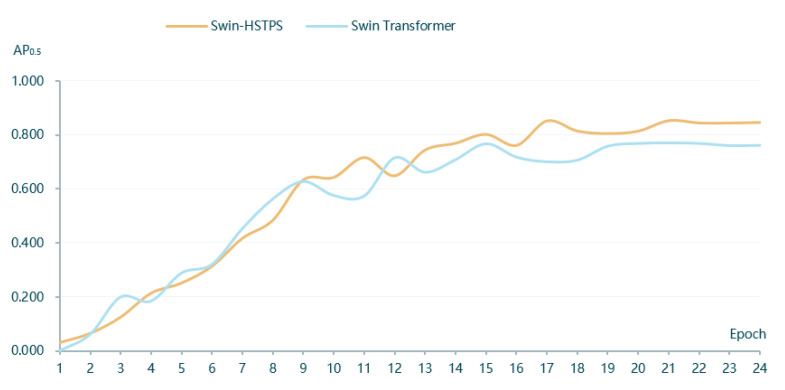
Average Precision comparison chart(AP_0.5_).

2. Prediction stability and convergence experiment

(1) The Swin-HSTPS and Swin Transformer target detection model training 24 batches of IoU 0.5 when the average precision is compared, as shown in Figure 4.

From the curve trend in Figure 4, it can be found that the average precision of the Swin-HSTPS in batches 1 to 12 has a faster growth, an approximate linear increase, and the fluctuation is small. Compared with the Swin Transformer, the average precision is close. At the same time, the average precision of the Swin-HSTPS in batches 13 to 24 is higher than that of the Swin Transformer. After the peak of 0.853 in batch 17, the average precision has been maintained at around 0.85. The Swin-HSTPS has better convergence than the Swin Transformer. The Swin-HSTPS model average precision is higher than the Swin Transformer model and the number of batches reaches 18 batches, and at the same time, the Swin-HSTPS has exceeded the Swin Transformer’s highest average precision value of 0.768 in batch 15 and batch 14 of the Swin-HSTPS, indicating that the Swin-HSTPS has been able to obtain average precision exceeding the Swin Transformer in batch 15 training; the Swin-HSTPS model can reach higher AP with fewer training rounds. This shows that the Swin-HSTPS has a better learning rate than the Swin Transformer training, and the overall training process can inherit or exceed the training efficiency of the Swin-HSTPS.

**Figure 5:**
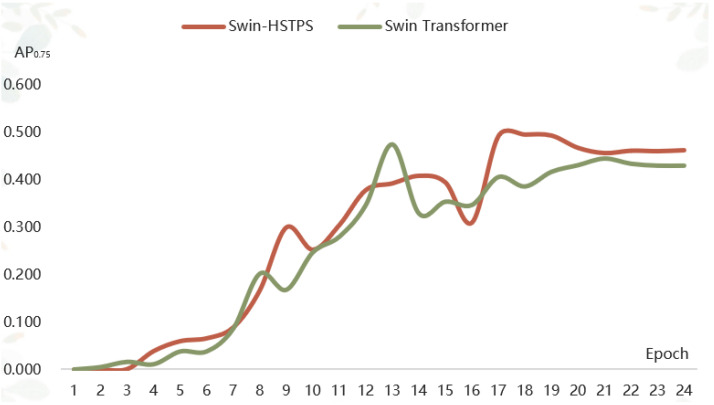
Average precision comparison chart (AP_0.75_).

(2) The Swin-HSTPS and Swin Transformer target detection model training 24 batches of IoU average precision of 0.75 for comparison, as shown in Figure 5.

From the curve trend in Figure 5, it can be found that the average precision of the Swin-HSTPS in batches one to nine has a faster growth, an approximate linear increase, and a small fluctuation. Compared with the Swin Transformer, the stability is stronger, and the average precision is slightly higher overall. At the same time, in addition to the 16 batches, the average precision of the Swin-HSTPS in batches 14 to 24 is basically higher than that of the Swin Transformer, and the average precision has been maintained at about 0.46 after reaching the highest point of 0.493 in batch 17. Compared with the Swin Transformer, the average precision of the Swin-HSTPS has exceeded 0.4. It was maintained at around 0.46, and the stability is better. The Swin-HSTPS model average precision is higher than the Swin Transformer model and the number of batches reaches 18 batches, indicating that the Swin-HSTPS has a better learning rate than the Swin transformer training, and the overall training process can inherit or exceed the training efficiency of the Swin-HSTPS.

**Figure 6:**
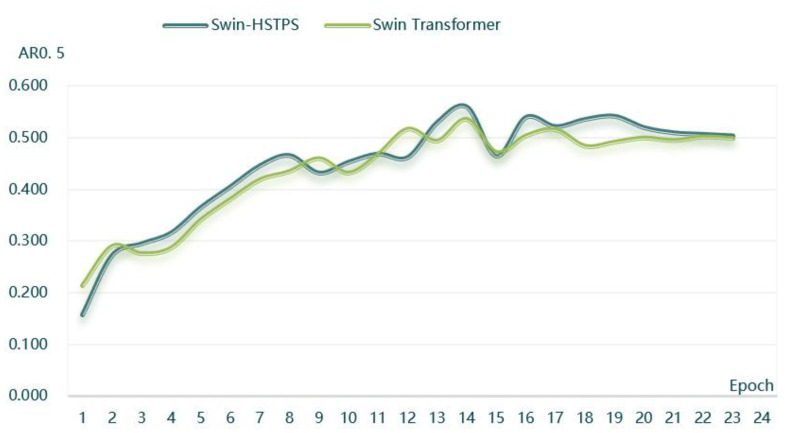
Average recall rate comparison chart (AR_0.5_).

(3) The Swin-HSTPS and Swin Transformer target detection model training 24 batches of average recall rate when the IoU is 0.5, as shown in Figure 6.

From the curve trend in Figure 6, it can be found that the average recall rate of the Swin-HSTPS from batches one to nine has a faster growth, an approximate linear growth, and less fluctuation. Compared with the Swin Transformer, it has stronger stability. At the same time, the overall average recall rate of the Swin-HSTPS during the training process is higher than that of the Swin Transformer, with a maximum of 0.56. The Swin-HSTPS has a stronger learning performance than the Swin Transformer model. The Swin-HSTPS model average recall is higher than the Swin Transformer model and the number of batches reaches 18 batches, indicating that the Swin-HSTPS has a better learning rate than the Swin transformer training, and the overall training process can inherit or exceed the training efficiency of the Swin-HSTPS.

**Figure 7:**
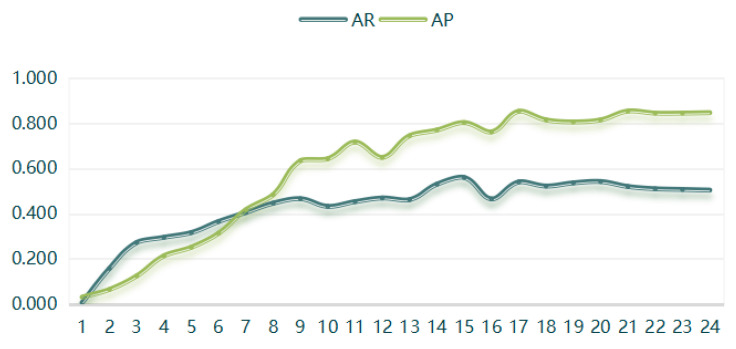
AR and AP comparison chart.

(4) The Swin-HSTPS target detection model training 24 batches of IoU is 0.5 when the average recall rate and average precision are compared, as shown in Figure 7.

From the curve trend in Figure 7, it can be found that the average recall rate and the average precision are positively correlated. At the same time, as the training batches increase, the average recall and average precision also increase, converging near the extreme. The Swin-HSTPS model has good stability.

In summary, from the training results, the Swin-HSTPS has a better training learning rate than the Swin Transformer, and the overall training process can inherit or exceed the training efficiency of the Swin Transformer.

### 4.3. Model Prediction Results

After training, the model files trained by the Swin-HSTPS and the Swin Transformer are used to predict the data set, respectively. This model is designed to realize automatic prediction algorithms from the reading of prediction data sets, data prediction, and storage of prediction results. Compared with the leaflet prediction mode of the Swin Transformer model, the batch prediction model of this model is more convenient. Through the prediction of the Swin-HSTPS and the Swin Transformer training models, each model has completed nearly 200 high-scoring remote sensing image predictions and completed aircraft and port predictions. The prediction results are compared below.

**Figure 8:**
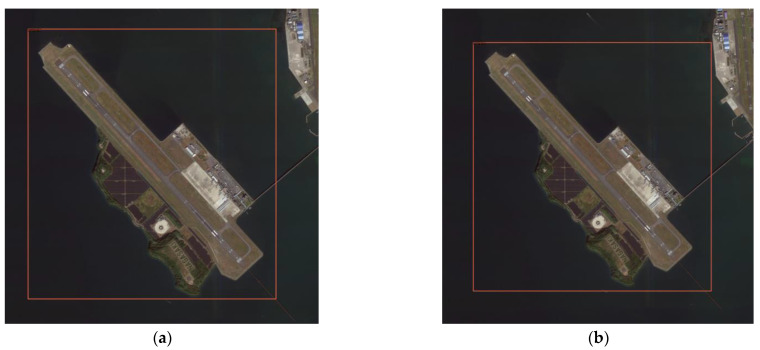
Airport prediction comparison: (**a**) Swin-HSTPS airport forecast results (confidence 0.99); (**b**) Swin Transformer airport forecast results (confidence 0.95).

**Figure 9:**
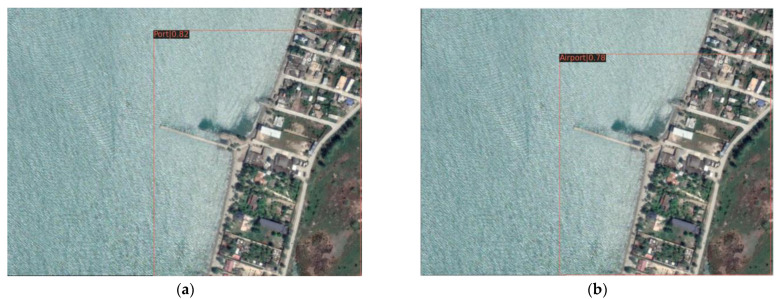
Port forecast comparison: (**a**) Swin-HSTPS port forecast result (confidence 0.82); (**b**) Swin Transformer port forecast results (confidence 0.78).

**Figure 10:**
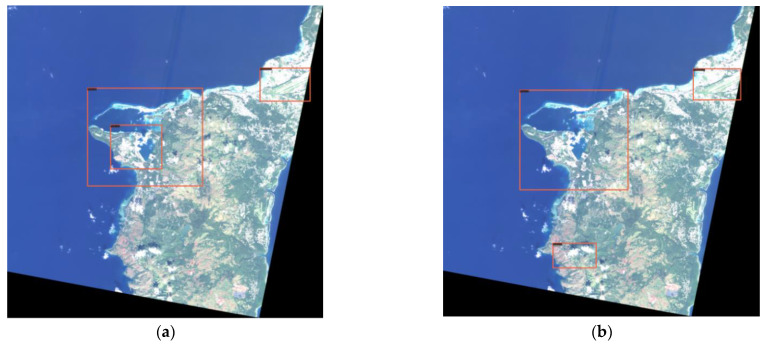
Comparison of multi-target prediction errors: (**a**) Swin-HSTPS’s multi-type target prediction results; (**b**) Swin Transformer’s multi-type target prediction results (incorrect mark in the lower left corner).

**Figure 11:**
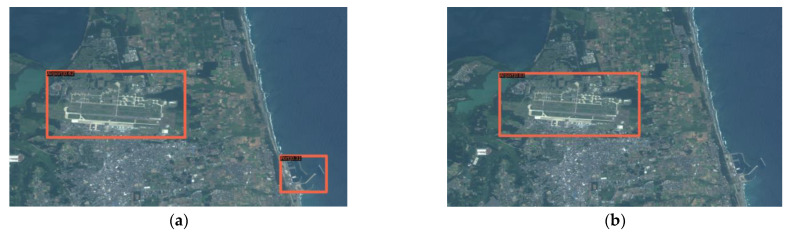
Multi-target prediction missed standard comparison: (**a**) Swin-HSTPS’s multi-type target prediction results; (**b**) Swin Transformer’s multi-type target prediction results (not marked in the lower right corner).

(1) High-scoring remote sensing images predict two types of buildings, airplanes and ports, and select two representative two sets of prediction results from 200 sheets, as shown in Figure 8 and Figure 9. It can be seen from the results that the Swin-HSTPS has a 3% higher confidence than the Swin Transformer for the two types of buildings. Through the statistics of about 200 images, more than 90% of the prediction results of the Swin-HSTPS are 2~3% higher than the Swin Transformer. From the comparison of the confidence of the prediction results, the Swin-HSTPS model has great advantages over the Swin Transformer. The Swin-HSTPS can improve the recognition of high-resolution remote sensing images for traffic port stations and provide technical support for automatic and precise positioning of high-resolution remote sensing images.

(2) The Swin-HSTPS and the Swin Transformer predict the accuracy of markers and make a relevant comparison. From Figure 10, it can be seen that the Swin Transformer incorrectly marked the port at the bottom of the image, and the Swin-HSTPS has a multi-level prediction of the port on the far left. It can be seen from Figure 11 that the Swin-HSTPS has marked one more port buildings than the Swin Transformer, and the Swin Transformer has missed the mark. The comparative analysis of the two sets of images shows that the Swin-HSTPS has a higher ability to predict and recognize buildings than the Swin Transformer. In addition, Figure 10 and Figure 11 both predict multiple types of target buildings. From the comparison of the accuracy of the prediction results, the Swin-HSTPS model has a better recognition accuracy than the Swin Transformer. It can basically find high-resolution remote sensing image traffic port station targets, reduce model recognition errors, and improve its positioning ability.

### 4.4. Discussion

In Section 4.1 to Section 4.3, through the comparative analysis of experimental results from several different angles, the Swin-HSTPS has achieved good results in terms of average precision, model learning rate, and prediction accuracy (prediction confidence) on high-resolution remote sensing satellite images.

From the results in Table 1, this paper selects the commonly used (α, β) fixed parameters of beta distribution for comparison experiments, and finds that the parameters α = 0.5 and β = 0.5 in MixUp obtain higher average precision in high-resolution remote sensing satellite images. The parameter is to adjust the degree of mixing of the data set (the degree to which the original data are retained). In order to improve the average precision of different types, or different images of interest in different types, or different images of interest, follow-up work will try to use the very fixed parameters of beta distribution (α, β) (fixed parameters are not used in this experiment) for related experiments. In addition, subsequent integration of better image enhancement algorithms, such as Retinex, will be applied in future experiments.

From the results in Table 2, the PReLU activation function setting parameter is 0.45 to obtain better average precision in high-resolution remote sensing satellite images, and this parameter adjusts the slope of the negative interval to reduce the disappearance of the gradient. Subsequent work will try to adjust the parameter to 0.5 or 0.25 as the progressive space in different image types or different objects of interest to test whether a better average precision can be achieved, but this parameter should be controlled to be less than one as far as possible to avoid overfitting. In addition, the main function of the activation function is to add the linear model to the nonlinear factor to complete the multi-classification problem. In order to improve the average precision of different types and different images of interest, operators can try to replace the PReLU activation function (such as RReLU, Elus, etc.).

## 5. Conclusions

Based on experiments on multi-source high-resolution remote sensing image data sets, from the training results, the maximum average precision of the Swin-HSTPS model is higher than that of the R-CNN, YOLOv5, and Swin Transformer. The experiment shows that the Swin-HSTPS model inherits the advantages of the Swin Transformer. The increased relative stability and robustness of the training batch has a slight advantage. At the same time, the target detection method for multi-source high-resolution remote sensing satellite imagery can train a higher average precision. From the prediction results, the Swin-HSTPS has higher recognition and positioning capabilities than the Swin Transformer.

In summary, the Swin-HSTPS model used in this article has more advantages and achieves the expected effect of the improved algorithm in this article. The Swin-HSTPS algorithm inherits the local self-attention mechanism in the non-overlapping window and the model structure integrated in a layered manner. At the same time, it combines the PReLU activation function and the MixUp data enhancement algorithm to enhance the image feature information and strengthen the information interaction of each pixel block, avoiding the disappearance of the gradient. The Swin-HSTPS algorithm better solves the problem of in-depth feature aggregation of high-resolution remote sensing image traffic port station supervision information, and improves the positioning accuracy of high-resolution remote sensing imagery.

## Figures and Tables

**Table 1:** The average accuracy of the Swin-HSTPS when MixUp varies with the value of (α, β).

(α, β)	Image Size	${\mathrm{AP}}_{50}^{\mathrm{box}}(\%)$	${\mathrm{AP}}_{75}^{\mathrm{box}}(\%)$
(5, 1)	224^2^	67.1	26.6
(2, 5)	224^2^	74.6	46.9
(1, 3)	224^2^	63	35
(0.5, 0.5)	224^2^	85.3	49.3
(2, 2)	224^2^	80.3	45.7

**Table 2:** The average accuracy of the Swin-HSTPS when PReLU varies with the value of a_i._

$a_{i}$	Image Size	${\mathrm{AP}}_{50}^{\mathrm{box}}(\%)$	${\mathrm{AP}}_{75}^{\mathrm{box}}(\%)$
0	224^2^	66.2	32.6
0.25	224^2^	76.3	41.8
0.4	224^2^	78.0	42.0
0.45	224^2^	85.3	49.3
0.5	224^2^	74.7	41.8
0.6	224^2^	72.5	41.6

**Table 3:** Index comparison table of each target detection model.

Model	Image Size	#Param.	FLOPs	${\mathrm{AP}}_{50}^{\mathrm{box}}$	${\mathrm{AP}}_{75}^{\mathrm{box}}$
R-CNN	224^2^	126.65 M	467.76 G	58.3	40.4
Swin Transformer	224^2^	135.778 M	814.719 G	77.1	47.5
Swin-HSTPS	224^2^	135.778 M	814.727 G	85.3	49.3
YOLOv5	224^2^	-	-	66.67	-
